# The Michelin red guide of the brain: role of dopamine in goal-oriented navigation

**DOI:** 10.3389/fnsys.2014.00032

**Published:** 2014-03-18

**Authors:** Aude Retailleau, Thomas Boraud

**Affiliations:** ^1^Sagol Department of Neurobiology, University of HaifaHaifa, Israel; ^2^Institut des Maladies Neurodegeneratives UMR 5293, University of BordeauxBordeaux, France; ^3^Institut des Maladies Neurodegeneratives UMR 5293, CNRSBordeaux, France

**Keywords:** dopamine, hippocampus, basal ganglia, spatial navigation, striatum

## Abstract

Spatial learning has been recognized over the years to be under the control of the hippocampus and related temporal lobe structures. Hippocampal damage often causes severe impairments in the ability to learn and remember a location in space defined by distal visual cues. Such cognitive disabilities are found in Parkinsonian patients. We recently investigated the role of dopamine in navigation in the 6-Hydroxy-dopamine (6-OHDA) rat, a model of Parkinson’s disease (PD) commonly used to investigate the pathophysiology of dopamine depletion (Retailleau et al., 2013). We demonstrated that dopamine (DA) is essential to spatial learning as its depletion results in spatial impairments. Our results showed that the behavioral effect of DA depletion is correlated with modification of the neural encoding of spatial features and decision making processes in hippocampus. However, the origin of these alterations in the neural processing of the spatial information needs to be clarified. It could result from a local effect: dopamine depletion disturbs directly the processing of relevant spatial information at hippocampal level. Alternatively, it could result from a more distributed network effect: dopamine depletion elsewhere in the brain (entorhinal cortex, striatum, etc.) modifies the way hippocampus processes spatial information. Recent experimental evidence in rodents, demonstrated indeed, that other brain areas are involved in the acquisition of spatial information. Amongst these, the cortex—basal ganglia (BG) loop is known to be involved in reinforcement learning and has been identified as an important contributor to spatial learning. In particular, it has been shown that altered activity of the BG striatal complex can impair the ability to perform spatial learning tasks. The present review provides a glimpse of the findings obtained over the past decade that support a dialog between these two structures during spatial learning under DA control.

## Introduction: of Parkinson’s disease, disorientation, dopamine and hippocampus

Parkinson disease (PD) has long been characterized as a motor disease (Agid, 1991). Emphasis has been stressed for long on the so-called triad of akinesia, rigidity and tremor. However classic drug and surgical therapies are now able to control more or less these symptoms, often at the cost of disabling side effects such as drug-induced dyskinesia (Boraud et al., 2001). Nevertheless, the increased autonomy acquired by the Parkinsonian patients unmasked cognitive symptoms previously underestimated (Sawamoto et al., 2002). It was previously thought that cognitive alteration was a late feature of the disease but, in fact, it occurs early in about 15–20% of the patients (Weintraub et al., 2011; Svenningsson et al., 2012). Amongst cognitive symptoms we can identify spatial disorientation, which has been described as an early landmark 25 years ago but left unexplored for a long time (Hovestadt et al., 1987; Taylor et al., 1989) despite its impact on life quality and public health problem (Crizzle et al., 2012).

Spatial cognition includes all the processes that allow animals to acquire process, memorize and use spatial information to perform goal-directed movements. Animals perceive space and extract pertinent information relevant to their spatial behavior using two types of sensory information: external cues supplied by the environment and internal cues supplied by their own movements. Navigation strategies are based on internal cues (path integration), external cues (taxon navigation) or on both (local navigation; O’Keefe and Conway, 1978; Gallistel, 1990; Trullier et al., 1997) Navigation could be represented in space by two systems of coordinates: an external coordinate system (allocentric representation) or an internal frame (egocentric representation; Berthoz, 1991; Poucet and Benhamou, 1997; Benhamou and Poucet, 1998). Several structures are considered to be important for building the neural representation of these spatial coordinates often called cognitive maps: amongst them the hippocampus is considered as the corner stone (O’Keefe and Dostrovsky, 1971; O’Keefe, 1979). Dopaminergic afferents to the hippocampal formation arise from both the ventral tegmental area (VTA, A10) and *substantia nigra pars compacta* (SNc, A8–A9) dopaminergic cell groups (Scatton et al., 1980). In addition, intra-hippocampal injections of D1 agonists and D2 antagonists improve memory (Packard and White, 1991; Wilkerson and Levin, 1999), while 6-Hydroxy-dopamine (6-OHDA) lesions of dopaminergic inputs to hippocampus induce spatial working memory deficits (Gasbarri et al., 1996). The dorsal part of Cornu Ammonis areas 3 (CA3), considered to be involved in the rapid acquisition of new memory (Kesner, 2007), is the target of dopamine (DA) projections from VTA and SNc (Scatton et al., 1980; Luo et al., 2011). DA innervation together with a higher liability of place fields as compared to those of CA1 (Barnes et al., 1990; Mizumori, 2006), makes CA3 a good candidate for the detection of the contextual significance of spatial features (Penner and Mizumori, 2012).

**Figure 1:**
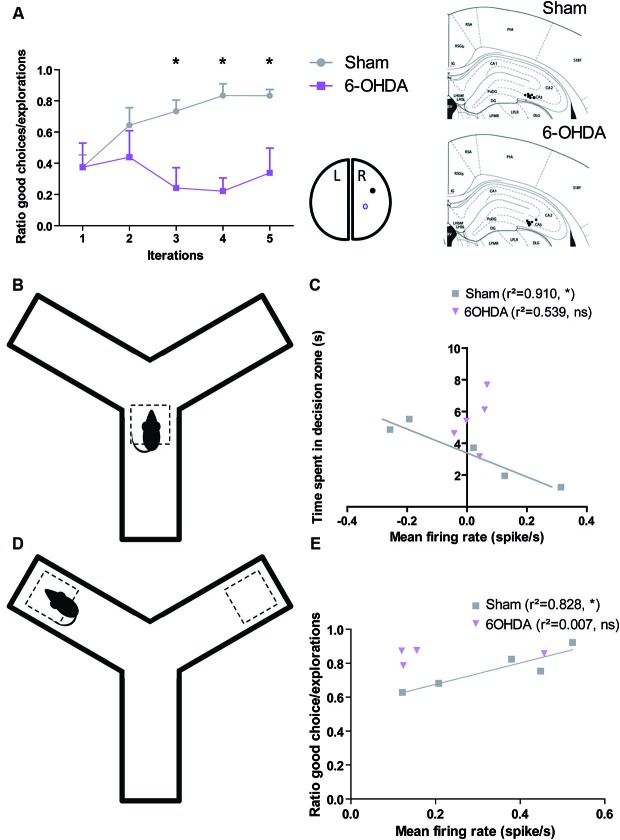
**Dopamine depletion modifies the encoding of decision process and reward location in CA3 (adapted form Retailleau et al., 2013). (A)** In a baited Y-maze, the rat has to choose one of the arms where the reward is located. Rat with a 6-OHDA lesion in the right medial forebrain bundle had difficulties to find the reward when it is in the right arm as it is shown by its low ratio of good choice (i.e., the baited arm)/the number of exploration. We correlated the behavior in the maze with population responses in the right CA3 (A, right). The time spent by the animal in the decision zone **(B)** is correlated with the population firing rate of CA3 neurons of Sham, but not 6-OHDA rats **(C)**. The ratio of good choice/total number of exploration is correlated with the population firing rate of CA3 neurons of Sham, but not 6-OHDA rats **(E)** when the animal is in the reward location **(D)**.

These facts raise the question of what happens in this brain structure when dopamine is depleted in Parkinsonian patient. We recently addressed this issue in the 6-OHDA rat, a model of PD commonly used to investigate the pathophysiology of dopamine depletion (Retailleau et al., 2013). We demonstrated that hippocampus played not only a role in the identification of locations in an environment and in the planning of the trajectories to goals and path selection as previously showed (Morris et al., 1990; Bannerman et al., 1995; Whishaw and Jarrard, 1995; Whishaw and Tomie, 1996; Hollup et al., 2001; Johnson and Redish, 2007; Rolls, 2007), but also in goal encoding. Behavioral analysis showed that sham rats are able to maximize their behavior in a baited Y-maze in order to increase the total amount of reward income over the course of the session. This behavior is correlated to an increase in the mean firing rate of neurons in CA3 at both decision point and reward location. Moreover, the lesion of the dopaminergic neurons of Substantia Nigra disrupted this ability and uncorrelated firing rate of CA3 neurons from decision and reward location (Figure 1).

However, the origin of these alterations in the neural processing of the spatial features needs to be clarified. It could result from a local effect: dopamine depletion disturbs directly the processing of relevant spatial information at hippocampal level. Alternatively, it could result from a more distributed network effect: dopamine depletion elsewhere in the brain (entorhinal cortex, striatum, etc.) modifies the way hippocampus processes spatial information. In this mini-review we address as survey of arguments in the literature that can support eachtheory.

## Influence of DA in the hippocampus

Dopamine from SNc and VTA provides a reward prediction error signal to the dorsal and ventral striatum (Schultz, 2006). It is tempting to assume that similar mechanisms are involved in the hippocampus itself. There are strong elements to support this assumption. Hippocampus is one of the rare brain areas where the 5 subtypes of DA receptors are found, D2-like (Brouwer et al., 1992) and D1-like families (Gingrich et al., 1992; Laurier et al., 1994; Sokoloff and Schwartz, 1995). In addition, intra-hippocampal injections of D1 agonists and D2 antagonists improves memory (Packard and White, 1991; Wilkerson and Levin, 1999), while 6-OHDA lesions of dopamine input to hippocampus result in spatial working memory deficits (Gasbarri et al., 1996). Exposure to a novel context increases hippocampal dopamine release (Ihalainen et al., 1999), which in turn facilitates long term potentiation (LTP) induction (Li et al., 2003; Lemon and Manahan-Vaughan, 2006). Detection of a context change by hippocampus can be used to update memory systems, which in turn may signal dopamine neurons to increase dopamine release. The subsequent increase in dopamine release in hippocampus seems to excite hippocampal neurons and so increase the duration of neural responses to glutamatergic input (Smialowski, 1987; Smialowski and Bijak, 1987) contributing as such to the increased stabilization of place fields typically observed as rats become familiar with new environmental conditions (Frank et al., 2004).

All of these data strengthened the hypothesis of a local effect of DA in the hippocampus, but there is a major drawback, which is that dopaminergic direct input to hippocampus, is very sparse and poorly described despite intensive search. As far as we know, we found only one study which described that a few fibers arise from both the VTA, A10 and SNc, A8–A9 dopaminergic cell groups (Scatton et al., 1980). But sparseness doesn’t mean inefficiency and the contrast with the reasonably high density of DA receptors in the hippocampus pleads for an actual functional effect.

## Basal ganglia and spatial navigation

Experimental approaches in rodents have provided behavioral evidence that pharmacological manipulations of the nucleus accumbens (NAc—part of the ventral striatum) impair acquisition and/or performance in spatial learning tasks (Annett et al., 1989; Wiener, 1993; Ploeger et al., 1994; Gal et al., 1997; Smith-Roe et al., 1999; Atallah et al., 2007; Ferretti et al., 2007). Packard and McGaugh’s seminal experiments (Packard and McGaugh, 1996) introduced the hypothesis that the striatum contributed to egocentric learning. However, recent data have generated controversy and show that the striatum is also involved in other aspects of spatial learning. Various studies examined the involvement of different sub-regions of the striatum in allocentric and egocentric learning (Voorn et al., 2004). Their results suggest that the Dorso Lateral Striatum (DLS) plays a role in egocentric spatial learning (Yin and Knowlton, 2004) based on cue-action association (van der Meer et al., 2010) and both the NAc and the Dorso Medial Striatum (DMS) plays a role in allocentric learning (Lavoie and Mizumori, 1994; Mizumori et al., 2004; Ferretti et al., 2007). The central role of the ventral striatum in spatial decision making has been confirmed by electrophysiological approach (Lansink et al., 2009; van der Meer et al., 2010; van der Meer and Redish, 2011).

These findings pave the way for integration between allocentric and egocentric process in the striatum. Striatum is a highly convergent structure where huge overlaps exist between the different functional territories (Parent and Hazrati, 1995). Thorn et al. (2010) recently demonstrated that DMS and DLS display contrasting patterns of activity during task performance that developed concurrently with sharply different dynamics. It suggests that this integration can be a cooperative (additive) process or a competition process that would contribute to multimodal decision making using mechanisms similar to those that have been modeled for action selection (Leblois et al., 2006; Guthrie et al., 2013).

## Influence of DA in the basal ganglia

The regulation of striatal input by tonic and phasic dopamine release is so well known and described that there is not enough space in this mini-review to detail and we report the reader to more comprehensive review (Goto et al., 2007; Schultz, 2007; Humphries et al., 2012). Briefly, dopamine presysnaptic button connect the base of dendritic spines of the Medium Spiny Neurons of the striatum. From this key position they control the effect of the other inputs connecting the head of the spine (Parent and Hazrati, 1993) and play a key role in synaptic plasticity.

The role of DA at striatal level in the control of spatial navigation has been evidenced both in its ventral and dorsal part. In the ventral part, evidences are accumulated since the late 1980s. Mogenson was the first to highlight that the hippocampal signal transmission to the pedunculopontine tegmental nucleus is regulated by dopamine receptors of the NAc (Mogenson et al., 1987). The integrative role of the NAc and subpallidal area in relaying hippocampal signals to the mesencephalic locomotor region in the brainstem was investigated electrophysiologically in urethane-anaesthetized rats. A behavioral study of the functional connections was also performed in freely moving rats. In the electrophysiological experiments, subpallidal output neurons to the pedunculopontine nucleus were first identified by their antidromic responses to electrical stimulation of the pedunculopontine nucleus. Hippocampal stimulation was then shown to inhibit orthodromically some of these subpallidal neurons. The inhibitory response was attenuated following microinjection of a dopamine D2 agonist, but not a D1 agonist, into the NAc. This suggests that signal transmission from the hippocampus to the subpallidal output neurons to the pedunculopontine nucleus is modulated by a D2 receptor-mediated mechanism in the NAc. Injections of N-methyl-D-aspartate into the ventral subiculum of the hippocampus resulted in a threefold increase in locomotor responses. Injection of a D2 agonist into the accumbens reduced the hyperkinetic response dose-dependently and suggests that D2 receptors regulate locomotor responses initiated by the hippocampal-accumbens pathway. These results provide evidence of limbic (e.g., hippocampus) influences on locomotor activity by way of NAc-subpallidal-pedunculopontine nucleus connections that may contribute to adaptive behavior.

Concerning the dorsal part (DMS and DLS), the role of DA in controlling spatial navigation has been partially demonstrated only much more recently (for review: Penner and Mizumori, 2012). What is known so far is that, DA depletion in the DLS interfere with strategy shifting, but no experiments has been conducted yet in order to assess if it induced disruption of the egocentric learning.

Thus despite it is highly probable that DA depletion in the striatum could explain the effect on spatial behavior, there is no definitive evidences yet.

## Towards an integrative representation

This quick review of the literature highlighted the multilevel implication of DA in the process of spatial information by the nervous system, however, it remains to determine at which level(s) the disruption of the dopaminergic function impacts spatial navigation in order to unravel the origin of spatial disorientation in PD patients (Hovestadt et al., 1987; Taylor et al., 1989).

The classical segregation between allocentric and egocentric learning seems too schematic and does not take into account the different modalities of perception of the environment. Hippocampus and striatum both contribute to encoding the spatial representation and seem to be involved in various modalities of perception. The hippocampus is related to the building of a spatial map (where am I?) but recent study evidenced that many place cells recorded from rats performing place or cue navigation tasks also discharged when they are at the goal location (Hok et al., 2007). The striatum provides reinforcing values to several aspects of the environment (where is my reward?), the ventral part to the pure localization aspects while the medial part encodes external cues. Meanwhile the dorsal striatum encodes the procedure (from here, which sequence of actions do I have to take in order to get my reward? (Retailleau et al., 2012). This taxonomy overlaps with the one recently proposed by Khamassi and Humphries (2012), which proposed that allocentric framework encompassed a model-based learning process while egocentric is more related to model free associative learning. It seems obvious that DA plays a key role in the striatum and that striatal DA depletion partly disrupts these processes. It is also highly probable that the rich density of DA receptors together with the response to reward location and decision processes we evidenced in CA3 (Retailleau et al., 2013) argue for also a local effect. So both mechanisms should be involved in the spatial disorientation observed in PD patients, but what remains to be established is in which proportion.

## How emerges a spatial strategy?

It has been shown that different spatial strategies can be used and that lesion of the hippocampus or one of the striatal territories may shift the dominant strategy used by the subject (Packard and McGaugh, 1996; Mizumori et al., 2004; Penner and Mizumori, 2012) It strongly suggests that the neural mechanism of the specific selection of one of these modalities is based on competition mechanisms.

**Figure 2:**
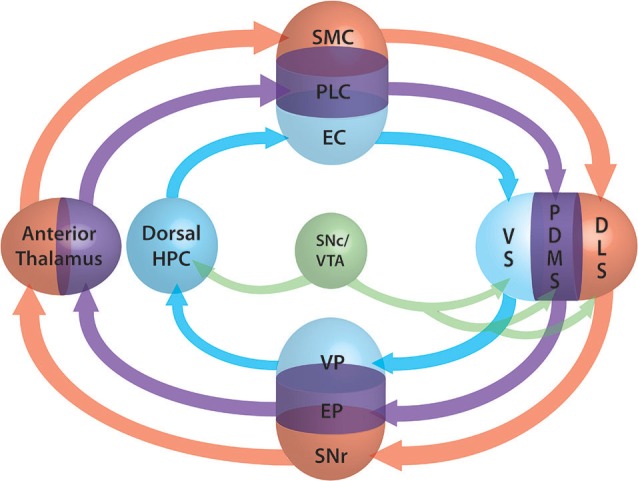
**Schematic representation of the three loops involved in spatial lnavigation**. The loops are embedded in the fronto-cortical structures (SMC: sensorimotor cortex, PLC: prelimbic cortex, EC: entorhinal cortex), the basal ganglia (BG) segregated roughly into ventral (VS: ventral striatum, VP: ventral pallidum), dorsomedial (DMS: dorsomedial striatum, EP: entopeduncular nucleus) and dorsolateral (DLS: dorsolat- eral striatum, SNr: substantia nigra pars reticulata) parts, the anterior thalamus and the dorsal part of the hippocampus (Dorsal HPC). Dopamine projections from the SNc and ventral tegmantal area (VTA) are shown in green. The three functional loops are shown in blue for allocentric navigation by localization, in purple for allocentric navigation by external cues and in red for egocentric navigation. Convergence at BG level allows the system to perform selection of one of the modalities through a competition mechanism similar to those of the action selection loop (adapted form Retailleau et al., 2012).

We demonstrated that action selection and decision making are emerging properties of the architecture of the frontal and dorsal part of the cortex-basal ganglia (BG) loop (Leblois et al., 2006; Guthrie et al., 2013). It makes it a perfect analogous of the actor in the actor-critic model of Sutton and Barto (1998), working under the supervision of a critic. Actor and critic are interfaced by DA at striatal level. The exact role of DA is still discussed and may be different in different structures (Khamassi and Humphries, 2012). While it is almost evident that it acts as error prediction signal in dorsal territories it may be simple reward signal elsewhere. In fact the neural substrate underlying the critic is still unknown and different sub-population of the actor structures can be involved (Lansink et al., 2009; Gläscher et al., 2010). For a comprehensive review of the involvement of the structures in different modalities of critic processing, we report the reader to Khamassi and Humphries (2012). We propose here to extend our model of the actor to the modalities of spatial navigation. The three modalities (external coordinates, external cues and internal coordinates) are supported by three different loops. The first loop passing through the ventral BG and the hippocampus, the second supported by the medial BG and thalamus respectively, and the third loop passing through the dorsal BG (Figure 2). The competition mechanism would allow the activation of one of the three modalities. During learning, dopamine would induce plasticity at various levels (ventral/medial or dorsal striatum, hippocampus) and modify the respective weight of each loop. According to the phase of learning and the reinforcing value of one of the aspects, one of the three networks would win the competition and therefore take over the control of the spatial navigation. This theory has the advantage of taking into account both the competitive and the collaborative aspects of their interactions and explains why, when all loops are functional, one can take over from another during the course of learning while, when one is disrupted, a different loop is able to take over the spatial navigation function. At the beginning of learning the system switches from one modality to another randomly due to the system noise level. Meanwhile, each system learns in parallel from the outcomes. From Packard and McGaugh (1996) work, we can infer that the more ventral system learns first and drives animal behavior after a moderate period of learning. After longer practice, the dorsal system is strengthened, takes over from the ventral system and drives the animal behavior. Inhibition of the stronger partner of the system (the ventral in the early phase, the dorsal in the later phase) allows the weaker system to take control of the behavior. According to the topography of the lesion, dopamine depletion as in PD may disrupt one or several of these systems. Where this disruption occurs and can it be compensated for by another system is still an open question to be investigated.

## Conflict of interest statement

The authors declare that the research was conducted in the absence of any commercial or financial relationships that could be construed as a potential conflict of interest.
